# Age-Related Shift in Neuro-Activation during a Word-Matching Task

**DOI:** 10.3389/fnagi.2017.00265

**Published:** 2017-08-10

**Authors:** Ikram Methqal, Jean-Sebastien Provost, Maximiliano A. Wilson, Oury Monchi, Mahnoush Amiri, Basile Pinsard, Jennyfer Ansado, Yves Joanette

**Affiliations:** ^1^Laboratory of Communication and Aging, Institut Universitaire de Gériatrie de Montréal, Montreal QC, Canada; ^2^Faculty of Medicine, University of Montreal, Montreal QC, Canada; ^3^Helen Wills Neuroscience Institute, University of California, Berkeley, Berkeley CA, United States; ^4^Centre de Recherche CERVO – CIUSSS de la Capitale-Nationale et Département de Réadaptation, Université Laval, Québec City QC, Canada; ^5^Hotchkiss Brain Institute, University of Calgary, Calgary AB, Canada; ^6^Department of Psychology, Université du Québec en Outaouais, Gatineau QC, Canada

**Keywords:** fMRI, word-matching task, executive processes, healthy aging, cognitive control profile, neuro-functional reorganization

## Abstract

Growing evidence from the neuroscience of aging suggests that executive function plays a pivotal role in maintaining semantic processing performance. However, the presumed age-related activation changes that sustain executive semantic processing remain poorly understood. The aim of this study was to explore the executive aspects of semantic processing during a word-matching task with regard to age-related neuro-functional reorganization, as well as to identify factors that influence executive control profiles. Twenty younger and 20 older participants underwent fMRI scanning. The experimental task was based on word-matching, wherein visual feedback was used to instruct participants to either maintain or switch a semantic-matching rule. Response time and correct responses were assessed for each group. A battery of cognitive tests was administrated to all participants and the older group was divided into two subgroups based on their cognitive control profiles. Even though the percentage of correct responses was equivalent in the task performance between both groups and within the older groups, neuro-functional activation differed in frontoparietal regions with regards to age and cognitive control profiles. A correlation between behavioral measures (correct responses and response times) and brain signal changes was found in the left inferior parietal region in older participants. Results indicate that the shift in age-related activation from frontal to parietal regions can be viewed as another form of neuro-functional reorganization. The greater reliance on inferior parietal regions in the older compared to the younger group suggests that the executive control system is still efficient and sustains semantic processing in the healthy aging brain. Additionally, cognitive control profiles underlie executive ability differences in healthy aging appear to be associated with specific neuro-functional reorganization throughout frontal and parietal regions. These findings demonstrate that changes in neural support for executive semantic processing during a word-matching task are not only influenced by age, but also by cognitive control profile.

## Introduction

Healthy aging is accompanied by changes in numerous cognitive abilities, with performance differences noted within and between cognitive domains (Valdois et al., 1990; Goh et al., 2012). For instance, slight age-related changes have been reported for cognitive abilities that involve semantic processing, which remains relatively stable across the lifespan (Burke and Shafto, 2004), unlike those abilities that have been shown to decline with age such as episodic memory, visual attention and inhibition (e.g., Park et al., 2002). However, these age-related cognitive changes are less extensive than one would expect, given age-related structural brain changes.

The preservation of cognitive performance in healthy aging is usually associated with adaptive changes in brain activity (Reuter-Lorenz and Cappell, 2008). One such neuro-functional change is the presence of more widespread activation involving both hemispheres in older adults, a phenomenon formalized in the HAROLD model (Hemispheric Asymmetry Reduction in Older Adults; Cabeza, 2002). In addition to inter-hemispheric neuro-functional symmetry, intra-hemispheric changes have been also described, such as the PASA phenomenon (Posterior–Anterior Shift with Aging; Davis et al., 2008). Interestingly, convergent findings from our laboratory and from other studies reflect that there is often an age-related activation shift from anterior to posterior regions to support cognitive performance (Ansado et al., 2013b; Lacombe et al., 2015). For instance, Ansado et al. (2013a) have reported an age-related additional parietal recruitment to cope with increasing cognitive demands during a load-dependent judgment task. Similarly, Oedekoven et al. (2013) have found a greater parietal activity contribution, which resulted in successful episodic memory retrieval among older individuals. Thus, the engagement of parietal regions at high-demanding tasks tends to reflect the neuro-functional reorganization within fronto-parietal networks that supports cognitive performance in healthy cognitive aging.

It is well known that one of the most important age-related changes in brain activation takes place in frontal regions that are known to be involved in executive (or cognitive) control processes (Paxton et al., 2008; Grady, 2012). Although one could assume, from the findings mentioned above, that these regions are not sufficient to explain all age-related differences in executive resources involved in maintaining task performance (Bouazzaoui et al., 2014; Collette and Salmon, 2014). Based on the most neuroimaging findings, changes in executive control functioning could be the first indicator of the brain’s adaptation strategy to insufficient neural resources in healthy aging by flexible neural reallocation (Reuter-Lorenz and Cappell, 2008; Adrover-Roig and Barceló, 2010). In one relevant neuroimaging study, Peelle et al. (2013) showed that older adults with better semantic processing tended to rely more on the prefrontal and inferior parietal regions for word-meaning judgment than younger adults or older adults with poorer semantic performance. These findings suggest that the executive control regions (i.e., frontal and parietal regions) constitute the neuro-functional basis for semantic performance maintenance in healthy aging.

From a cognitive perspective, cognitive control supports a variety of executive processes defined as the ability to maintain and update information in working memory and to switch from current information to the adoption of new information, which involve a higher level of executive control (Miyake et al., 2000; Braver et al., 2003; Adrover-Roig et al., 2012). There has been, however, relatively little investigation on age-related neuro-functional changes of neural patterns relevant to executive control processes in semantic tasks.

The relative preservation of semantic processing can be defined by the degree of control over maintaining and/or switching among different types of semantic word relationships (Nagel et al., 2008; Noonan et al., 2010; Maintenant et al., 2011; Whitney et al., 2012). Growing evidence is emerging from functional neuroimaging studies that have considered the interactions between the executive control and language networks (Whitney et al., 2011; Noonan et al., 2013; Lambon Ralph et al., 2016). Consistent findings from these studies reveal the extent of executive control network activation for semantic performance in language comprehension tasks. These large neural networks underpinning semantic and executive processes consist of inferior prefrontal, anterior cingulate, inferior parietal and posterior temporal cortices, as well as the cerebellum (Bookheimer, 2002; Noppeney et al., 2004; Binder et al., 2009). For instance, prefrontal regions supporting executive control processes are specifically active for the effective use of relevant semantic knowledge as well as when manipulation of semantic relationships is required during retrieval and selection among semantically related competitor words (Thompson-Schill et al., 1997; Wagner et al., 2001; Badre and Wagner, 2007; Maintenant et al., 2013). In addition to this prefrontal involvement, inferior parietal regions were also consistently activated across semantic tasks with high level of executive control (Noonan et al., 2010; Whitney et al., 2012). However, it is unclear whether these age-related changes in the activation of cognitive control networks sustain the semantic processing of words or if such activation reveals some dynamic processes. Overall, we could claim that studying executive aspects of semantic words processing is particularly appropriate for exploring possible neuro-functional reorganization with age.

Considering the fronto-parietal network involvement in semantic tasks requiring executive control processes (Binder and Desai, 2011; Noonan et al., 2013; Peelle et al., 2013), the first goal of this study was to explore, behaviorally and neuro-functionally, the age-related activation changes in fronto-parietal regions that underlie executive aspects of semantic processing. More specifically, an original word-matching task was developed based on the executive requirements of the Wisconsin Card Sorting Test (WCST) and was adapted for use in fMRI protocols (Monchi et al., 2001). This task requires the flexible use of semantic relationship (or rules) supported by two executive processes: (a) maintain rule; and (b) switch rule. The first process requires participants to maintain a given semantic rule through working memory updates, while the second one requires a shift from one rule to another. The latter is related to higher-level of executive control relative to the former.

Given the neuro-functional changes that occur concomitantly with the relative preservation of semantic ability in healthy aging (Ansado et al., 2013a; Peelle et al., 2013; Lacombe et al., 2015), we expected that older adults show greater activation in the inferior parietal regions, relative to younger adults. More precisely, these age-related neuro-functional changes were expected at the higher-level of executive control process necessary for the switch rule rather than for the maintain rule. Finally, it was expected that behavioral performance differences, measured by response times and correct responses, would be correlated with brain activation changes for both groups.

A current issue in cognitive aging studies is the reorganization of executive processes that occurs in contribution to age-related changes performance in complex executive tasks. In the past decade of cognitive aging studies, many researchers have supported the idea of the non-unitary nature of executive functions in healthy aging (Hull et al., 2008; Adrover-Roig et al., 2012), unlike those who have focused on a single and common executive system in aging (de Frias et al., 2006). These claims might further be viewed as extending work of Miyake et al. (2000) by supporting the notion that age-related changes in executive performance could be better explained by diversity in executive functions represented by at least two distinct executive subcomponents, though they are not completely independent. These executive processes consist of updating and maintaining information in working memory, and shifting between mental sets. Such distinction has also been found at the level of the neuro-functional organizations between updating and shifting processes, the former process supported by the activation of prefrontal regions, while the latter being mainly associated with parietal regions engagement (e.g., Collette et al., 2006; Braver et al., 2009; Herd et al., 2014; Kopp et al., 2014).

These executive processes are defined as distinct executive subcomponents that are differentially used by older adults. Some findings have further highlighted that higher level of global cognitive functioning would be associated with largely separable executive control processes (Adrover-Roig and Barceló, 2010; Collette and Salmon, 2014). In this context, de Frias et al. (2006) have reported that high cognitive functioning in older adults is related to highly differentiated executive control processes rather than unitary process. Moreover, Adrover-Roig et al. (2012) have found neuro-functional changes that are associated with the level of cognitive control in older adults. In that study, older adults with low level of cognitive control showed less activation in frontal regions indicating inefficiency in updating/maintaining processing compared to older adults with high level of cognitive control. As the existence of changes in executive function among older adults is supported by distinct executive control processes underpinned by neurally distributed networks, the second goal of this study was to explore whether different cognitive control profiles in aging would correspond to specific neuro-functional reorganization patterns.

Based on different cognitive measures that encompass executive (or cognitive) control processes, we aimed to identify if (a) at a behavioral level, individual differences in executive performance could be triggered by different cognitive control profile (updating-specific and shifting-specific) among older adults; and (b) at a neuro-functional level, neuro-functional reorganization patterns are associated to specific cognitive control profiles. More specifically, the possibility that central cognitive control processes may be linked to specific cognitive profiles was examined in elderly people. It was expected that older adults with a shifting-specific profile would rely more extensively on parietal regions, while the updating-specific profile would recruit more frontal regions to maintain the performance to a given task. Finally, it was also expected that there would be a correlation between behavioral performance (response times and correct responses) and task-induced brain activation changes in each cognitive profile within an elderly group.

## Materials and Methods

### Participants

Twenty healthy older adults aged between 63 and 80 and 20 younger adults whose ages ranged from 19 to 35 were recruited from a pool of volunteers at the Centre de Recherche de l’Institut Universitaire de Gériatrie de Montréal (CRIUGM). All participants were native French speakers and all were right-handed (scores greater than +95) as assessed by the Edinburgh Handedness Inventory (Oldfield, 1971). All had normal or corrected-to-normal vision; none had any history of major neurological disease, psychiatric illness, head injury, stroke, substance abuse, learning disabilities, or any problems that could interfere with behavior testing. Prior to the neuro-imaging session, all participants were also given a battery of neuro-psychological tests during a single 90-min session which included: screening of global cognitive function (The Montreal Cognitive Assessment, MoCA; Nasreddine et al., 2005); the inhibition measure (Stroop Test; Stroop, 1935); the flexibility measure (Trail Making Test, TMT A and B; Reitan, 1955); working memory measure (forward and backward Digit Span, WAIS III; Wechsler, 1981); several measures of ability to select a rule, maintain it, and switch to a new rule are from Burgess and Shallice (1997), for the Brixton test and Nelson (1976), for the Wisconsin Card Sorting Test (WCST); and semantic fluency as represented by the total number of words produced in 2 min for the category Animals (Cardebat et al., 1990). **Table 1** provides a detailed description of the raw cognitive measures as well as a statistical comparison of group means. Furthermore, the older adults’ cognitive scores (not shown in **Table 1**) were within the average range according to all psychometric standardized data, suggesting normal cognitive functioning within the older adult group. All participants gave written informed consent to the protocol, which was approved by the Institut universitaire de gériatrie de Montréal Human Ethics Committee and by the Regroupement Neuroimagerie/Québec (RNQ). This committee follows the guidelines of the Civil Code of Quebec, the Tri-Council Policy Statement of Canada, the Declaration of Helsinki, and the code of Nuremberg. Finally, in order to clearly identify subgroups of older participants according to their cognitive control profile, classification was based on their *z*-score for neuropsychological tests.

**Table 1:** Means (M) and standard deviations (SD) of the demographic and neuropsychological variables of all participants (*n* = 40).

	Younger (*n* = 20)	Older (*n* = 20)		
	*M* (*SD*)	*M* (*SD*)	*F*(1,38)	*p*-values
Age	24.85 (3.85)	69.45 (4.54)	1129.02	0.001
Gender (F: M)	16:4	17:3	0.603	0.714
Education (years)	17.95 (2.52)	18.85 (2.88)	1.01	0.301
Edinburgh inventory	95%	96%	0.89	0.122
MoCA	28.6 (1.53)	28.7 (1.03)	0.058	0.81
Stroop C	49.95 (6.88)	62.2 (9.12)	22.95	0.001
Stroop W	39.25 (4.02)	45.30 (5.82)	14.61	0.001
Stroop C–W	83.05 (13.42)	114.65 (22.29)	29.5	0.001
TMT A	17.40 (4.35)	27.60 (8.22)	23.98	0.001
TMT B	41.60 (11.77)	62.82 (16.29)	22.24	0.001
Digits forward	10.5 (1.67)	9.65 (1.75)	2.46	0.125
Digits backward	8.45 (2.03)	6.8 (1.73)	7.6	0.009
Brixton (errors)	1.15 (1.03)	1.45 (1.05)	0.82	0.37
WCST (errors)	0.88 (1.19)	1.05 (1.27)	0.41	0.527
Semantic Fluency	39.70 (8.27)	28.85 (8.1)	17.55	0.001

### Characterization of Older Subgroups

Five executive *z*-scores for each older participant were entered into hierarchical cluster analysis with CLUSTAN (Aldenderfer and Blashfield, 1984). Using the Clustan Graphics program (version 5.27), case classification was based on the squared Euclidean distance as a coefficient of similarity, and on the Ward method of classification (Ward, 1963). The *k*-means clustering procedure of relocation was then applied to ensure that the two-cluster solution was stable. This procedure allowed for the identification of two possible natural older subgroups of participants, based on their performances on five executive measures (TMT B/A, digits backward, number of errors on the Brixton test, number of errors on the WCST, number of words produced correctly for the semantic fluency task). This grouping was confirmed *a posteriori* using an independent-samples *t*-test, which revealed that the two subgroups (henceforward referred to as the updating-profile and shifting-profile groups) differed significantly on five neuro-psychological tests (see part B).

The updating-profile group scored significantly higher on the backward digit span and Trail Making Test (Part B) than on the WCST, Brixton, and semantic fluency tests. Conversely, the shifting-profile group scored significantly higher on the WCST, Brixton, and semantic fluency tests than on the backward digit span and Trail Making Test (Part B). Thus, the latter group’s executive performance relied more on their shifting ability than on updating/working memory, which the former group depended on more.

A cluster analysis approach was also performed on the group of younger participants. However, the results provided no clear indication of a given cognitive profile associated with a sub-group of participants. Thus, for younger participants, behavioral data were analyzed as a group.

### Experimental Procedure

**FIGURE 1:**
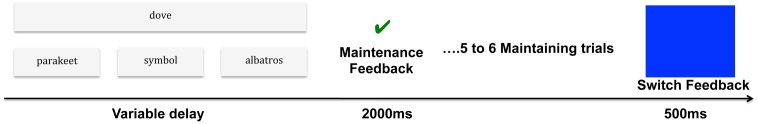
Experimental procedure of the word-matching task. In this example, each participant performed a task in which a target word presented at the top of the screen, dove (‘*colombe*’) had to be paired with one of three reference words, presented at the bottom, according to three possible semantic relationships: (a) typically related word (co-hyponyms) parakeet (‘*perruche*’); (b) atypically related word (co-hyponyms) albatross (‘*albatros*’); and (c) functionally related words (F) symbol (‘*symbole*’). The sorting period was followed by a maintenance feedback signal (green check mark displayed for 2000 ms) indicating that participants should maintain the same semantic rule as in the previous trial. After 5 or 6 maintaining trials, the rule changed (blue screen displayed for 500 ms) and participants had to discover the new classification rule and maintain it. As a control condition, a target card (e.g., AAAA) would be matched with the same sequence (e.g., aaaa) among three reference cards: aaaa, bbbb, cccc.

The word-matching task used in this study was based on the computerized WCST developed and adapted to fMRI by Monchi et al. (2001) and Simard et al. (2011). The word-matching task was administered using stimulus presentation software (Media Control Function; Digivox, Montréal, QC, Canada). Throughout the task, three reference cards based on three semantic rules were presented in a row at the bottom of the screen, displaying moderately, atypical, and functionally related words (see **Figure 1** for example). In each trial, a new target card was presented in the middle of the screen above the reference cards; it displayed a highly typical word. Participants must then match the target card with one of the reference cards based on moderately typical, atypical, or functional relatedness. Participants used a joystick to select among the three reference words, pressing left, right, or upward to select the reference word on the left, on the right, or in the middle, respectively (the description of selection stimuli is reported in Supplementary Data Sheet 1).

The word-matching task trials contained two periods: matching and feedback.

• The matching period started with the presentation of a new target card (highly typical word). The participant then chose one of the three reference words by using one of the three joystick directions. The length of each matching period depended on the participant’s response times, which varied between 1470 and 4690 milli-seconds (ms) for this task. The period ended when the participant provided a selection response.
• The feedback period was indicated by a blue screen, which lasted for 500 ms and started as soon as a first correct match was made. Feedback was conveyed through a specific cue lasting for 2000 ms. An incorrect match was indicated by a red cross, whereas a correct match was indicated by a green check mark, which informed participants that the current matching rule was the correct one and that they should maintain the same rule as in the previous trial (see **Figure 1** for experimental procedure).
• In addition, there were control trials during which the target card was represented by a series of letters (e.g., AAAA), which was identical with one of the three reference cards (e.g., aaaa, bbbb, cccc). These trials involved pairing a target with an identical reference card (alphabetic association: AAAA with aaaa). No rule changes occurred in the control condition and control feedback indicated a correct or incorrect match.

All participants had one fMRI session, which consisted of four runs. Blocks of each of the four trials (the three semantic rule trials and the control trial) were presented in pseudo-random order four times per run. The rules changed without warning and the new correct rule would be applied and maintained until the participant achieved five to six consecutive correct matching trials (maintaining a rule if shown a green check mark) or had to switch it (if presented with a blue screen as feedback). It is worth mentioning that no participant reported learning the sequence regularity or having deduced the frequency of the changing rule. The control block consisted of eight trials. For each participant, the total number of trials per run changed according to performance, which depended on the number of errors. The participants were fully trained on the word-matching task by performing a block of conditions outside the scanner. Each participant needed to reach a performance level of 90% correct matching trials and have less than 5% of set-loss and perseverative errors before moving on to the scanning session.

The stimuli were presented via an LCD projector onto a mirror placed in front of the participant in the MRI scanner. Stimuli were outlined in black against a white background to improve visual contrast. All words were displayed horizontally at the top of the screen and were centered on a computer screen placed 50 cm away from the participant. The target word was placed in a larger rectangle and subtended a visual angle of 26.6° horizontally and 13.8° vertically. All words were presented in 28-point Arial font, and reference words were placed in three small rectangles 1.3 cm apart from each other.

For this study, we explored, exclusively, executive processing during the word-matching task. All correct (5–6) consecutive matching trials, after the maintenance feedback period and the correct trial after switch feedback, were taken into account for behavioral and imaging analysis, as were the correct control matching trials. To ensure that the rule was successfully acquired after rule-matching change (related to the search for a correct rule), we removed the first correct trial after switch feedback.

## Data Analysis

### Behavioral Data

Two behavioral measures were also collected: response times and correct responses (defined as 5 or 6 consecutive correct matching trials after maintenance and switch feedback). Intergroup analyses were performed using SPSS 18.0 software for Mac (IBM SPSS Statistics 18). A comparison ANOVA was done between the two groups (younger and older) for each executive component (henceforth, matching after maintenance feedback is referred to as *maintain rule* and matching after switch feedback as *switch rule*) and between these executive components for each group (younger and older). For these analyses, the response times for control matching trials were subtracted from those for matching trials after maintain rule in order to account for age-related decline in motor speed (Fristoe et al., 1997; Martins et al., 2014). In addition, errors were analyzed for each group (younger vs. older) and a one-way ANOVA was carried out.

Results for response times and correct responses were divided into two parts; the first part (A) was based on a comparison between groups (younger vs. older), and the second part (B) was based on a comparison within the older group. This latter part of the study was exploratory.

### fMRI Scanning

#### Image Acquisition

Participants were scanned at the Unité de Neuroimagerie Fonctionnelle of the Institut de Gériatrie de Montréal using a 3T Siemens Trio Magnetom MRI scanner (Siemens AG, Erlangen, Germany). The structural scan was a high-resolution T1-weighted 3D-MPRAGE, sagittal plane acquisition, field of view (FOV) = 256 mm, and matrix size = 256 × 256. In addition, we acquired functional images [T2^∗^ weighted, TR = 2500 ms, TE = 30 ms, 36 slices parallel to the anterior and posterior commissure (AC-PC) line, slice thickness = 3.5 mm with 3.5 mm^3^ isotropic voxels, distance factor 0% (gap = 0 mm), Flip-angle = 90°, matrix = 64 × 64]. Each 252-volume functional run lasted 10.30 min; four such runs were acquired for each participant. The stimulus presentation and the scanning were synchronized at the beginning of each run. To minimize head movement during scanning, cushions were placed between the subject’s head and the coil.

#### fMRI Data Analysis

FMRI Expert Analysis Tool (FEAT) Version 5.98, part of the FSL analysis package (FMRIB’s Software Library, Version 4.1.4^1^), was used to conduct image pre-processing procedures. We corrected for head motion using MCFLIRT (FMRIB’s motion correction linear image registration tool; Jenkinson et al., 2002), and also used the fsl_motion_outliers script to detect and remove any volumes with excessive head motion. Non-brain tissue was removed using BET (Brain Extraction Tool; Smith, 2002). Grand-mean intensity normalization was applied to the 4D dataset from each run based on multiplicative scaling factor. We applied a Gaussian kernel of 6 mm FWHM for spatial smoothing, and for temporal filtering, a high-pass filter was applied to remove low-frequency noise using Gaussian-weighted least-squares straight-line fitting (1/60 Hz). Temporal auto-correlation was corrected by using pre-whitening as implemented by FILM (FMRIB’s improved linear model). Functional images of each participant were co-registered to structural images in native space, and structural images were normalized to Montreal Neurological Institute (MNI) standard space using FSL’s MNI Avg 152 T1 2 mm × 2 mm × 2 mm. The same transformation matrices used for structural-to-standard transformations were then used for functional-to-standard space transformations of co-registered functional images.

The FEAT module in FSL was used for first level analysis. An event-related design was used to model the fMRI data, allowing for inference based on contrast. We included five different event types in the design matrix: typical, functional, and atypical maintain rules; switch rules; and control trials. The maintain rule period was defined on the basis of the time period, for which each length varied between trials depending on the participant’s response time. This period started with the presentation of a new trial and ended only when the participant provided a selection response. The maintain rule period was convolved with a double-gamma hemodynamic response function (HRF). The switch rule period was defined as a shift event based on the second correct trial after switch feedback, during which the participant had to discover and apply the new matching rule, convolved with a double-gamma HRF. The aim was to separate correct maintain and switch-rule periods as well as control trials. Motion regressors generated by MCFLIRT were then included as confound covariates. A first-level GLM analysis was carried out separately for each run, followed by a second-level fixed-effects analysis. We then combined these analyses across all participants in group-level analysis (Higher-level) using a mixed effects analysis controlling for variation within and between participant groups, using FLAME (FMRIB’s Local Analysis of Mixed Effects). For age-group comparison (younger vs. older), statistical results were at a threshold voxel significance level of Z > 2.3, and a whole-brain-corrected cluster significance threshold of *p* < 0.05. To explore the effect of cognitive control profile on activation changes in the older group, an exploratory study was conducted. For the older subgroup’s exploratory study, all the steps in the analysis were as described above. However, Flames 1 and 2 were added at a higher level of analysis due to the small sample size of the older subgroups. For the same reason, the statistical results were at a threshold voxel significance level of *Z* > 1.96 and a whole-brain-corrected cluster significance threshold of *p* < 0.05 for the main-effect of each subgroup. For subgroup difference analysis, due to a limited sample size, a more liberal threshold at *p* < 0.001 was set. In this manner, a literature-guided hypothesis was tested for activity differences specifically located in frontoparietal regions, while ensuring that no other significant clusters were found in the brain.

To investigate regional differences of BOLD (blood-oxygen-level dependent) signal changes in the areas that showed significant activation, we used a region of interest (ROI) approach. Each ROI centered on a peak level of significant activation produced by a group average of the statistical maps. For the between-group comparison (younger vs. older), these ROIs were in the right posterior prefrontal cortex [BA 6/8/44; *x* = 37; *y* = 18; *z* = 52] and in the left inferior parietal cortex [BA 39/40; *x* = -33; *y* = -61; *z* = 39]. For between older subgroups (updating-profile vs. shifting-profile): within the left dorsolateral prefrontal cortex [BA 9/46; *x* = -42; *y* = 10; *z* = 35] and the left inferior parietal cortex [BA 39/40; *x* = -33; *y* = -61; *z* = 39] were considered as ROIs. These ROIs (identified in our participants) have been widely reported in cognitive control tasks in previous studies (Rypma et al., 2006; Badre and D’Esposito, 2007; Nagel et al., 2008; Niendam et al., 2012; Whitney et al., 2012; Noonan et al., 2013). ROI masks were generated with a 4 mm-radius sphere centered on the peak voxel coordinate within each significant cluster. The mean BOLD signal change of the ROIs was extracted separately for each participant from the maintain rule and switch rule. Finally, we conducted a correlation analysis for each age group and also for each older subgroup to investigate potential patterns of relation between behavioral performance (response times, correct responses) and BOLD signal changes during the word-matching task. Additionally, the Fisher *r*-to-*z* transformation was also applied to calculate a value of *z* that can be applied to assess the significance of the difference between two correlation coefficients found, in one case, between younger and older groups and, in the other, older subgroups separated by cognitive profile.

## Results

### Part A: Age-Related Neuro-Functional Reorganization

#### Behavioral Performance

Response times in maintain rule proved to be significantly longer in the older adult group than in the younger group [*M*_older_ = 2741 ms, *SD* = 481; *M*_younger_ = 2265 ms, *SD* = 705; *F*(1,38) = 6.219, *p* = 0.017]. The same was true in the switch rule [*M*_older_ = 2974 ms, *SD* = 793; *M*_younger_ = 2365 ms, *SD* = 965; *F*(1,38) = 4.74, *p* = 0.036] and control condition [*M*_older_ = 1330 ms, *SD* = 166; *M*_younger_ = 1048 ms, *SD* = 207; *F*(1,38) = 22.44, *p* = 0.001]. For the maintain rule condition, when control response times were taken into account, older adults showed no significant difference in response times compared to younger adults [*F*(1,38) = 1.704, *p* = 0.20] (**Table 2**).

**Table 2:** Behavioral performance (response times and correct responses) in word-matching task for 20 younger and 20 older adults.

	Younger	Older		
	Mean (*SD*)	Mean (*SD*)	*F*(1,38)	*p*-values
**Response times (in milliseconds)**				
Experimental condition				
Maintain rule	1217 (542)	1411 (383)^∗^	1.704	0.20
Switch rule	2365 (965)	2974 (793)	4.74	0.036
Control condition	1048 (207)	1330 (166)	22.44	0.001
Total correct	95.26 (5.30)	93.11 (7.51)	1.093	0.302
responses (in %)				

*SD, standard deviation. ^∗^Control condition response time was subtracted from only maintain rule condition*.

A 2 × 2 ANOVA was performed to explore the effects of age group (younger vs. older) and executive component (maintain rule vs. switch rule) on response times. There was a marginal effect of age group [*F*(1,38) = 3.64, *p* = 0.064], with response times slower in the older adults than in the younger adults. The interaction between age and executive component was also significant [*F*(2,38) = 7.32, *p* = 0.010]. The main effect analysis revealed a significant effect of the executive component [*F*(1,38) = 313.25, *p* = 0.001], showing that maintain rule took less time than switch rule. More specifically, for the maintain rule, planned comparisons did not reveal any significant effect of age [*M*_older_ = 1411 ms, *SD* = 383; *M*_younger_ = 1217 ms, *SD* = 542; *F*(1,38) = 1.704, *p* = 0.20], whereas the older adults showed significantly longer response times in the switch rule condition than the younger adults. It is worth noting that there was no difference between older and younger participants in total correct responses or in the word-matching task [*F*(1,38) = 0.693, *p* = 0.41] regardless of feedback type, indicating that both younger and older adults performed well.

#### Imaging Results

The aim of this study was to investigate the impact of age on neuroimaging patterns during a word-matching task. The brain activation pattern was described for maintain rule and switch feedback compared to the control condition. Given the relevance of executive aspects in the word-matching task used in this study, we combined the three semantic relationships. We compared the average BOLD signal obtained during maintain rule and switch rule with the control matching condition. Intergroup analyses were also performed.

##### Maintain rule vs. control matching

As predicted, neuroimaging analyses revealed the involvement of cognitive control networks during the word-matching task in both groups (younger and older). The younger group showed significant activation in the right dorsolateral prefrontal cortex (BA 9/46), the left ventrolateral prefrontal cortex (BA 44/45), the bilateral insula (BA 41), the left lateral premotor cortex (BA 6), the left posterior prefrontal cortex (junction of BAs 6, 8, and 44), the bilateral superior parietal cortex (BA 7), and the left inferior parietal cortex (BA 39).

The older adults showed significant activation in the left ventrolateral prefrontal cortex (BA 44/45), the bilateral insula (BA 41), the bilateral posterior prefrontal cortex (junction of BAs 6, 8, and 44), the dorsolateral prefrontal cortex (BA 9/46) bilaterally, the left lateral premotor cortex (BA 6), the left inferior temporal cortex (BAs 37 and 20), the bilateral superior parietal cortex (BA 7), the left inferior parietal cortex (BA 39), the left occipital cortex (BA 18), and the right cerebellum.

**FIGURE 2:**
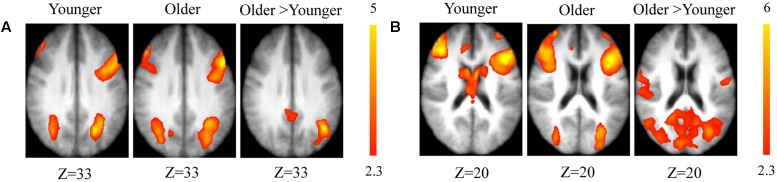
**(A)** Brain activation for maintain rule minus control condition. The younger group (cf. left). The older group (cf. middle). The comparison between the older adults and the younger adults (cf. right). The color scale represents the *Z* statistic. *Z*-values correspond to the coordinate of the axial plane. **(B)** Brain activation for switch rule minus control condition. The younger group (cf. left). The older group (cf. middle). The comparison between the older adults and the younger adults (cf. right).

The comparison between groups showed significantly greater activation in the older adults than in the younger ones in the left and the right hemispheres in the posterior cingulate cortex (BA 31), the right inferior temporal cortex (BA 37), the left inferior cortex (BA 40), the cerebellum bilaterally, the right occipital cortex (BA 18), and in the bilateral caudate nucleus. Comparison of younger minus older adults showed no significant difference (**Table 3** and **Figure 2A**; for younger and older see Supplementary Table S1).

**Table 3:** Maintain rule minus control matching.

		MNI peak (mm)				
Cluster	Anatomical areas	*x*	*y*	*z*	*Z* score	Voxel
	**Younger > Older**					
	**Older > Younger**					
1	Left caudate nucleus	-6	18	-6	4.05	20788
	Right caudate nucleus	6	16	-1	4	
2	Left inferior parietal cortex (area 40)	-42	-60	31	4.05	23136
	Right posterior cingulate cortex (area 31)	18	-55	18	3.58	
	Left posterior cingulate cortex (area 31)	-16	-58	21	3.17	
3	Left cerebellum	-27	-83	-31	4	32824
	Right cerebellum	40	-65	-23	3.7	
	Right inferior temporal cortex (area 37)	46	-57	-22	3.69	
	Right occipital cortex (area 18)	11	-98	-11	3.64	

##### Switch rule vs. control matching

As predicted, stronger activation was found in both groups in the switch rule relative to control matching. The younger group showed significant activation in the right dorsolateral prefrontal cortex (BA 9/46), the left supplementary motor area (BA 6), the left ventrolateral prefrontal cortex (BA 44/45), the right posterior prefrontal cortex (junction of BAs 6, 8, and 44), the right anterior cingulate cortex (BA 32), the bilateral superior parietal cortex (BA 7), the inferior parietal cortex bilaterally (BA 39), the left occipital cortex (BA 18), and the bilateral caudate nucleus.

The older adults showed significant bilateral activation in the frontopolar cortex (BA 10), the right dorsolateral prefrontal cortex (BA 9/46), the left supplementary motor area (BA 6), bilateral insula (BA 41), the left posterior prefrontal cortex (junction of BAs 6, 8, and 44), the bilateral lateral prefrontal cortex (BA 6), the inferior parietal cortex bilaterally (BA 40), and the right superior parietal cortex (BA 7).

The comparison between groups showed significantly greater activation in the older adults than in the younger adults in the left supplementary motor area (BA 6), the left inferior parietal cortex (BA 39/40), and the right cerebellum. Comparison of younger minus older adults showed no significant difference (**Table 4** and **Figure 2B**; for younger and older see Supplementary Table S2).

**Table 4:** Switch rule minus control matching.

		MNI peak (mm)				
Cluster	Anatomical areas	*x*	*y*	*z*	*Z* score	Voxel
	**Younger > Older**					
	**Older > Younger**					
1	Left SMA (area 6)	-1	-5	48	3.41	16016
2	Left inferior parietal cortex (area 39/40)	-59	-28	31	3.33	16065
3	Right cerebellum	30	-68	-40	4.29	28680

##### Switch rule vs. maintain rule

When the switch rule was compared with the maintain rule, the younger group showed significant activation in the left frontopolar cortex (BA 10), the left anterior cingulate (BA 32), the right dorsolateral prefrontal cortex (BA 9/46), the right posterior prefrontal cortex (junction of BAs 6, 8, and 44), the right inferior parietal cortex (BA 40), the bilateral superior parietal cortex (BA 7), and the left occipital cortex (BA 18).

The older adults showed significant activation in the left frontopolar cortex (BA 10), the left dorsolateral prefrontal cortex (BA 9/46), the left supplementary motor area (BA 6), the left inferior parietal cortex (BA 40), the superior parietal cortex bilaterally (BA 7), and the left occipital cortex (BA 18).

**FIGURE 3:**
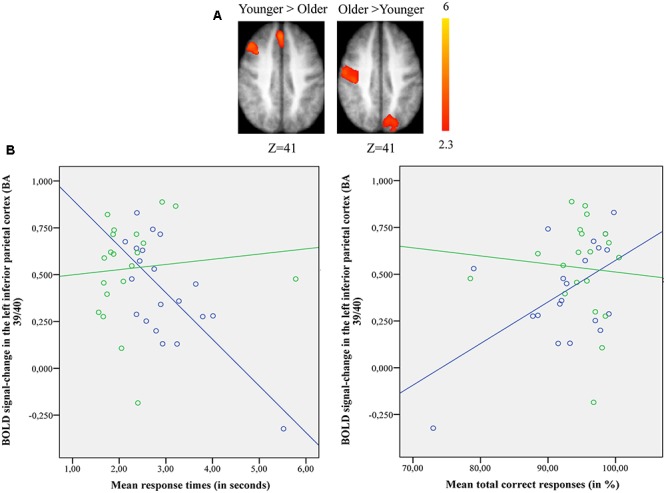
**(A)** Brain activation for performing switch rule relative to maintain rule for the younger adults compared to the older in the right posterior prefrontal cortex (cf. right) and in the left inferior parietal cortex in the older adults compared to the younger adults (cf. left). The color scale represents the *Z* statistic. *Z*-values correspond to the coordinate of the axial plane. **(B)** Correlation between activation in the left inferior parietal cortex and behavioral measures for younger (blue circles) and older adults (green circles). The left plot represents response times and the right plot the total correct responses for two groups. Note that correlation between behavioral measures and brain activity change in the right posterior prefrontal cortex did not reach any significant difference for two groups (not shown in this figure).

The comparison between groups showed significantly more activation in the older adults than in the younger adults in the left posterior cingulate cortex (BA 31), the left inferior parietal cortex (BA 39), the superior parietal cortex (BA 7), and the left occipital cortex (BAs 18 and 19). The comparison of younger minus older adults showed significantly more activation in the right supplementary motor cortex (BA 6) and the right posterior prefrontal cortex (junction of BAs 6, 8, and 44) in the younger adults (**Table 5** and **Figure 3A**; for younger and older see Supplementary Table S3).

**Table 5:** Switch rule minus maintain rule.

		MNI peak (mm)				
Cluster	Anatomical areas	*x*	*y*	*z*	*Z* score	Voxel
	**Younger > Older**					
1	Right SMA (area 6)	1	37	37	3.95	13342
	Right posterior prefrontal cortex (junction of 6, 8, and 44)	37	18	52	3.81	
	**Older > Younger**					
1	Left occipital cortex (area 19)	-26	-78	26	3.51	11648
	Left occipital cortex (area 18)	-11	-75	28	3.22	
2	Posterior cingulate cortex (area 31)	-12	-51	16	3.24	21623
	Left superior parietal cortex (area 7)	-12	-74	37	3.19	
	Left inferior parietal cortex (area 39)	-47	-67	28	3.13	

The comparison of brain activity patterns in the two age groups revealed more pronounced activity in the left inferior parietal cortex for the older adults and in the right posterior prefrontal cortex for the younger adults only when the maintain rule was subtracted from the switch rule. To explore the age-related neuro-functional relevance of these regions involved in the executive processes underlying the word-matching task, we first tested for an interaction effect before exploring the simple effects. Finally, we did a correlation analysis between older and younger participants’ performance and brain activity within these regions.

##### ROI BOLD signal and performance in older and younger groups

The results of 2 (group) × 2 (executive component) mixed effects ANOVA on BOLD signal in the left inferior parietal cortex revealed a significant main-effect of group [*F*(1,38) = 5.923, *p* = 0.020] and executive component [*F*(1,38) = 32.72, *p* = 0.001], but no group × executive component interaction [*F*(2,38) = 0.478, *p* = 0.49]. However, planned comparison showed significant differences between younger and older adults. The BOLD signal change in the left inferior parietal cortex was significantly greater for older adults when compared to younger adults only for switch rule [*F*(1,38) = 62.88, *p* = 0.001]. No significant main-effect of group [*F*(1,38) = 2.169, *p* = 0.149] or executive component [*F*(1,38) = 0.389, *p* = 0.537] was observed in the right posterior prefrontal cortex, with no group × executive component interaction [*F*(2,38) = 3.140, *p* = 0.073].

Pearson’s correlation analysis conducted between behavioral performance (response times, correct responses) and BOLD signal changes in the left inferior parietal cortex [BA 39/40; *x* = –33; *y* = -61; *z* = 39] for younger and older adults (**Figure 3B**) showed significant negative correlation with response times (*r* = -0.72, *p* = 0.001) and significant positive correlation with correct responses (*r* = 0.55, *p* = 0.011) in the older group. However, the younger adults showed no significant correlation with response times (*r* = 0.098, *p* = 0.68) or correct responses (*r* = -0.077, *p* = 0.74). The difference in correlation coefficients between the BOLD signal changes in the left inferior parietal cortex and the response times was statistically significant (*z* = -2.94, *p* = 0.0003) between the two groups (younger vs. older), as well as for the correct responses (*z* = 2.03, *p* = 0.042). There was no significant correlation between BOLD signal changes in the right posterior prefrontal cortex [BA 6/8/44; *x* = 37; *y* = 18; *z* = 52] and behavioral performance for younger (response times: *r* = 0.028, *p* = 0.90; correct responses: *r* = 0.04, *p* = 0.85) and older adults (response times: *r* = -0.43, *p* = 0.06; correct responses: *r* = 0.19, *p* = 0.40) (not shown in **Figure 3B**). The statistical difference between the two correlation coefficients for the two groups (younger vs. older) was not significant for response times (*z* = 1.42, *p* = 0.15) or for correct responses (*z* = -0.44, *p* = 0.65).

### Part B: Cognitive Control Profiles and Neuro-Functional Reorganization in Older Adults

#### Behavioral Performance

**FIGURE 4:**
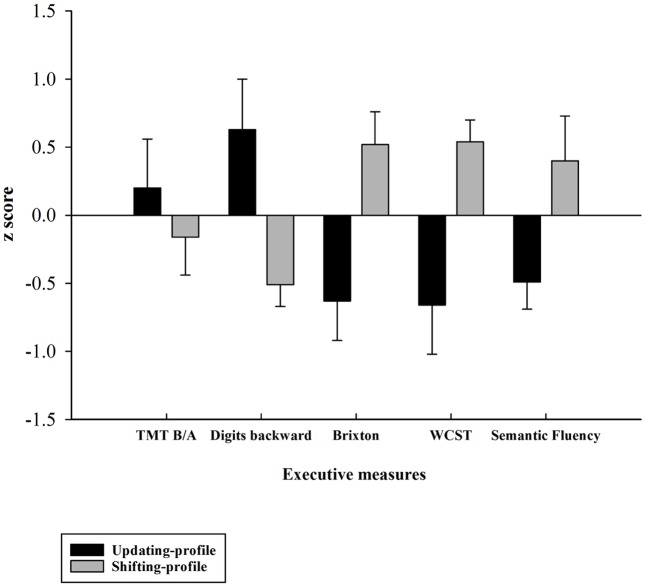
*Z*-executive scores of the two older subgroups (updating-profile vs. shifting-profile) based on five executive measures: TMT B/A, digits backward, number of errors on the Brixton test, number of errors on the WCST, number of words produced correctly on the semantic fluency task). Tests for which increasing values in the original scores indexed lower performance were reversed in sign so that increasing values always reflected higher performance. Errors bars are represented by standard error mean values (SEM).

The two older subgroups’ (**Figure 4**) behavioral performance (correct responses and response times) on the word-matching task was equivalent. A comparison between the two older subgroups (updating-profile vs. shifting-profile) for maintain rule and switch rule was performed using an independent-group *t*-test. The difference between the two older subgroups was not significant for maintain rule [*t*(18) = -0.641, *p* = 0.52] or for switch rule [*t*(18) = -0.440, *p* = 0.66] (**Tables 6**, **7**).

**Table 6:** Means (*M*) and standard deviation (*SD*) on the demographic and neuropsychological variables of the sample of older adults (*n* = 20) divided into two subgroups: updating-profile (*n* = 9) and shifting-profile (*n* = 11).

	Updating-profile	Shifting-profile		
	*M* (*SD*)	*M* (*SD*)	*t*(1.18)	*p*-values
Age	70.77 (4.96)	68.36 (4.08)	1.194	0.248
Education (years)	18.44 (2.12)	19.18 (3.45)	-0.558	0.584
MoCA	28.77 (0.97)	28.63 (1.12)	0.298	0.769
Stroop C	65 (3.1)	59.90 (2.63)	-1.262	0.223
Stroop W	46.33 (1.99)	44.45 (1.75)	-0.705	0.490
Stroop C–W	112.88 (7.04)	116.09 (7.27)	0.314	0.757
TMT A	24.55 (1.76)	30.09 (2.87)	1.552	0.138
TMT B	52.11 (4.04)	71.54 (4.26)	3.259	0.004
TMT B/A	2.25 (0.30)	2.55 (0.23)	0.799	0.435
Digits forward	9.88 (0.58)	9.45 (0.54)	0.539	0.596
Digits backward	7.88 (0.63)	5.99 (0.28)	3.039	0.007
Brixton (errors)	2.11 (0.30)	0.90 (0.25)	-3.057	0.007
WCST (errors)	1.88 (0.45)	0.36 (0.20)	-3.267	0.004
Semantic fluency	24.88 (1.65)	32.09 (2.68)	-2.161	0.044

**Table 7:** Behavioral performance (response times and correct responses) on the word-matching task for two older subgroups: updating-profile (*n* = 9) vs. shifting-profile (*n* = 11).

	Updating-profile	Shifting-profile		
	Mean (*SD*)	Mean (*SD*)	*t*(1.18)	*p*-values
**Response times (in milliseconds)**				
Experimental condition				
Maintain rule	2677 (453)	2818 (529)	-0.641	0.529
Switch rule	3062 (994)	2901 (625)	-0.440	0.665
Control condition	1341 (163)	1315 (178)	0.344	0.735
Total correct responses (in %)	92.25 (7.67)	93.11 (6.37)	0.271	0.789

#### Imaging Results

##### Maintain rule vs. control matching

**FIGURE 5:**
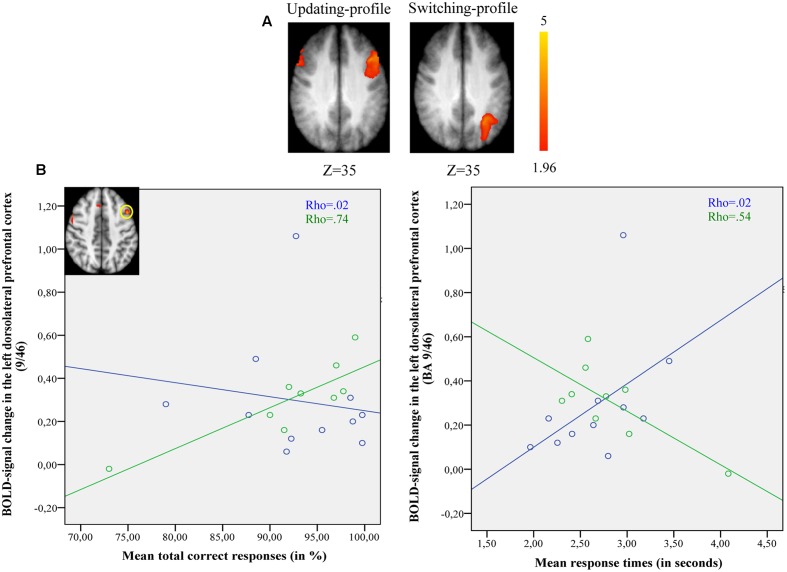
**(A)** Brain activation for maintain rule minus control matching. The updating-profile group (cf. left). The shifting-profile group (cf. right). The color scale represents the *Z* statistic. *Z*-values correspond to the coordinate of the axial plane. **(B)** Correlation the left dorsolateral prefrontal cortex [BA 9/46; *x* = –42; *y* = 10; *z* = 35] and behavioral measures for the updating-profile group (green circles) and shifting-profile group (blue circles). The left plot represents the total correct responses and the right plot mean response times. On the upper left, the updating-profile group shows significant activation in the left dorsolateral prefrontal cortex (BA 9/46) compared to shifting-profile group.

The updating-profile group showed significant activation in the left ventrolateral prefrontal cortex (BA 47/12), left dorsolateral prefrontal cortex (BA 9/46), the left posterior prefrontal cortex (junction of BAs 6, 8, and 44), the left lateral premotor cortex (BA 6), and the cerebellum bilaterally. The shifting-profile group showed significant activation in the left inferior parietal cortex (BA 40), the left superior parietal cortex (BA 7), and the left inferior temporal cortex (BA 20). The comparison between the two groups showed more activation in the updating-profile group than in the shifting-profile group within the left dorsolateral prefrontal cortex (BA 9/46). The reverse inter-group comparison showed no significant difference (**Table 8** and **Figure 5A**; for each older subgroups see Supplementary Table S4).

**Table 8:** Maintain rule minus control matching.

		MNI peak (mm)				
Cluster	Anatomical areas	*x*	*y*	*z*	*Z* score	Voxel
1	Updating-profile > Shifting-profile					
	Left dorsolateral prefrontal cortex (area 9/46)	-45	14	45	2.86	96
	Shifting-profile > Updating- profile					

##### Switch rule vs. control matching

**FIGURE 6:**
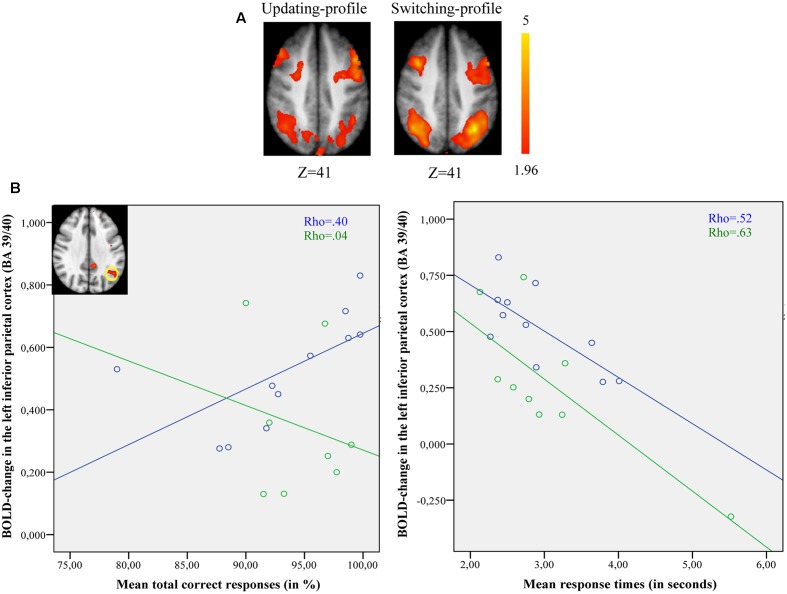
**(A)** Brain activation for switch rule minus control matching. The updating-profile group (cf. left). The shifting-profile group (cf. right). **(B)** Correlation between the left inferior parietal cortex [BA 39/40; *x* = –33; *y* = –61; *z* = 39] and behavioral measures for the updating-profile group (green circles) and shifting-profile group (blue circles). The left plot represents the total correct responses and the right plot the mean response times. On the upper left, the shifting-profile group shows significant activation in the left inferior parietal cortex (BA 39/40) compared to updating-profile group.

The updating-profile group showed significant activation in the right dorsolateral prefrontal cortex (BA 9/46), the left posterior prefrontal cortex (junction of BAs 6, 8, and 44), the right lateral premotor cortex (BA 6), the left superior parietal cortex (BA 7), the occipital cortex (BAs 18 and 19), and the cerebellum bilaterally. The shifting-profile group showed significant activation in the left frontopolar cortex (BA 10), the bilateral posterior prefrontal cortex (junction of BAs 6, 8, and 44), the right dorsolateral prefrontal cortex (BA 9/46), the left ventrolateral prefrontal cortex (BA 44/45), the right lateral premotor cortex (BA 6), the right superior parietal cortex (BA 7), the left inferior parietal cortex (BA 39/40), and the right cerebellum (**Table 9** and **Figure 6A**; for each older subgroup see Supplementary Table S5). The comparison between the two subgroups showed more activation in the shifting-profile group than the updating-profile group within the left inferior parietal cortex (BA 39/40). The reverse inter-group comparison showed no significant difference.

**Table 9:** Switch rule minus control matching.

		MNI peak (mm)				
Cluster	Anatomical areas	*x*	*y*	*z*	*Z* score	Voxel
	Updating-profile > Shifting-profile					
1	Shifting-profile > Updating-profile					
	Left inferior parietal cortex (area 39/40)	-45	-57	35	2.36	104

The comparison of brain activity patterns of the two older subgroups revealed more pronounced activity in the left dorsolateral prefrontal cortex for the updating-profile group, only during maintain rule. However, the shifting-profile group showed more pronounced activity in the left inferior parietal cortex (BA 39/40), only during switch rule. To explore the functional changes and age-related differences in cognitive control profiles, an interaction effect was tested before exploring simple effects. Finally, a correlation analysis was performed between the updating-profile group and the shifting-profile group’s behavioral performance and BOLD signal, within functionally relevant regions.

##### ROI BOLD signal and performance in the two older subgroups

The results of 2 (cognitive profiles) × 2 (executive component) mixed effects ANOVA on BOLD signal in the left dorsolateral prefrontal cortex revealed a significant main-effect of cognitive profile [*F*(1,18) = 4.41, *p* = 0.051] and executive component [*F*(1,18) = 9.129, *p* = 0.008], but no cognitive profile × executive component interaction [*F*(2,18) = 0.172, *p* = 0.68]. Similarly, significant main-effects of cognitive profile [*F*(1,18) = 6.151, *p* = 0.023] and executive component [*F*(1,18) = 25.48, *p* = 0.001] were observed in the left inferior parietal cortex in the absence of a cognitive profile × executive component interaction [*F*(2,18) = 1.27, *p* = 0.275]. However, planned comparison of BOLD signal change in the left dorsolateral prefrontal cortex showed significant difference between updating-profile and shifting-profile. The BOLD signal change in the left dorsolateral prefrontal cortex was significantly greater for the updating-profile when compared to the shifting-profile, only for the maintain rule (*p* = 0.028). Conversely, the BOLD signal changes in the left inferior parietal cortex were significantly greater for the shifting-profile when compared to the updating-profile, only for the switch rule (*p* = 0.038).

Spearman’s correlation analysis was conducted between behavioral performance (response times, correct responses) and BOLD signal changes in the left dorsolateral prefrontal cortex [BA 9/46; *x* = -42; *y* = 10; *z* = 35] and in the left inferior parietal cortex [BA 39/40; *x* = -33; *y* = -61; *z* = 39] for both older subgroups (updating-profile and shifting-profile).

During maintain rule, relative to control matching (**Figure 5B**), the correlation between BOLD signal changes in the left dorsolateral prefrontal cortex and correct responses was significantly positive for the updating-profile group (*r* = 0.83, *p* = 0.005), while for the shifting-profile group there was no significant correlation (*r* = -0.25, *p* = 0.486). The difference in correlation coefficients between BOLD signal changes in the left dorsolateral prefrontal cortex and correct responses was significant (*z* = 2.63, *p* = 0.008) between updating-profile and shifting-profile groups. Furthermore, there was no significant correlation between the BOLD signal change in the left dorsolateral prefrontal cortex and response times in either of the two older subgroups (updating-profile: *r* = -0.50, *p* = 0.055; shifting-profile: *r* = 0.60, *p* = 0.085). The difference between correlation coefficients was significant for updating-profile and shifting-profile groups (*z* = -2.18, *p* = 0.002).

Comparing switch rule to control matching (**Figure 6B**), there was no correlation between BOLD signal changes in the left inferior parietal cortex and correct responses (*r* = -0.14, *p* = 0.736) for the updating-profile, while positive correlation was observed for the shifting-profile group (*r* = 0.82, *p* = 0.002). Note: one outlier participant from the updating-profile group (extremely long response time) was removed from correlation analysis. The difference between the two correlation coefficients was significant for updating-profile and shifting-profile groups (*z* = -2.28, *p* = 0.022).

Furthermore, there was no correlation between the BOLD signal change in the left inferior parietal cortex and response times (*r* = -0.48, *p* = 0.22) for the updating-profile group, while a negative correlation was observed for the shifting-profile group (*r* = -0.72, *p* = 0.023). The difference between the two correlation coefficients was not significant for the updating-profile or the shifting-profile groups (*z* = 0.71, *p* = 0.47).

## Discussion

The aim of this study was to explore the age-related, neuro-functional basis for executive semantic processing of words. It is known that healthy aging is associated with neuro-functional reorganization that maintains cognitive performance. Herein, this investigation evaluated the effects of healthy aging and cognitive changes on executive function, neuro-functional activation, and behavioral measures during a word-matching task. To do so, a new word-matching task was employed that was based on the WCST and was adapted for fMRI by Monchi et al. (2001) and Simard et al. (2011). Results demonstrated that the shift in age-related brain activation from frontal to parietal regions is another form of neuro-functional reorganization, which sustains executive processes during a word-matching task. In addition, differences in cognitive control profile during aging appeared to be mediated by specific neuro-functional reorganization, which maintains task performance. Taken together, these results demonstrate functional changes during a word-matching task related to age and cognitive control profile. Further, correlations were identified between behavioral task performance and changes in brain activity within the relevant frontoparietal regions. These findings are discussed below.

### Age-Related Neuro-Functional Reorganization

As predicted, and consistent with the literature (Jefferies and Lambon Ralph, 2006; Whitney et al., 2011; Noonan et al., 2013), the cognitive control network was found to be involved in executive aspects of semantic processing, including the lateral prefrontal cortex, the anterior cingulate cortex, the parietal and temporal cortices, which are responsible for executive processing in the semantic domain that overlaps general executive processing. These findings are consistent with the recent neurobiology of language model, which proposes that successful language processing is based on the interaction between the neural mechanisms underlying cognitive control and the semantic system (Badre et al., 2005; Nagel et al., 2008). The brain activity patterns that emerged during the word-matching task revealed some age-related changes that appear to support better performance, even though the response time of older adults (compared to younger adults) was significantly longer after switch than maintenance feedback. It should be noted that differences in age-related response times were not systematically observed when control-matching performance was taken into account through a motor-speed measure. There was an age-related decline in performance of complex executive tasks such as the WCST, but controlling for a decrease in perceptual-motor speed in a task involving perception of stimuli followed by a simple motor response – the control condition (i.e., alphabet pairing in our study) – significantly reduced this performance decline (Fristoe et al., 1997; Martins et al., 2014). Taken together, our findings along with evidence from cognitive aging studies suggest that age-related differences in the reliance on high-level executive control processes, as it related to switching process (i.e., switch rule) during a word-matching task, may undergo greater age-related neuro-functional changes than the maintenance processes (i.e., maintain rule).

Regarding the maintain rule, bilateral frontal activation (**Table 3**) observed in older adults, compared to more lateralized activations in younger adults, suggests that the posterior prefrontal cortex (junction of BAs 6, 8, and 44) and the dorsolateral prefrontal cortex (BA 9/46) were recruited more when older participants maintained and updated rule classifications in working memory. The presence of posterior prefrontal involvement during different cognitive control tasks has been attributed to flexible cognitive performance (Brass and Von Cramon, 2004; Derrfuss et al., 2005). However, regarding these neuro-functional patterns, the differential involvement of the two lateral prefrontal regions (dorsal and ventral parts) in cognitive control may mediate different kinds of control. Indeed, Spreng et al. (2010) have suggested that older adults show reduced maintenance of information mediated by the left ventrolateral prefrontal cortex when compared to greater activation in the dorsolateral prefrontal cortex, which is more involved in strategic control.

When older adults were compared with younger ones, activations were noted in the posterior cingulate cortex, inferior parietal cortex, inferior temporal cortex, occipital cortex, and the superior and inferior portions of the cerebellum. Such activations are consistent with the pattern of activity seen during a set of semantic tasks, thought to require activation of specific conceptual knowledge features (Cristescu et al., 2006). The activations reported in the cerebellum are compatible with studies that have shown such activation in cases where high-level language processing is required in the context of tasks that require frontal areas to support lexico-semantic strategies (Walter and Joanette, 2007; Murdoch, 2010), as well as the storing and maintenance of information in working memory (Bellebaum and Daum, 2007). Overall, these changes in neuro-functional patterns support the hypothesis that older adults rely more on posterior regions in order to efficiently process semantic rules during word matching. The more demands are placed on their semantic knowledge, the more their strategic semantic processes involve the posterior regions, that underlie successful cognitive performance (Hazlett et al., 1998; Wierenga et al., 2008; Ansado et al., 2013b; Lacombe et al., 2015).

As predicted, switch rule compared to control matching was associated with bilateral frontal and parietal activations in both younger and older adults, although activation was greater in older adults (**Figure 2B**). These regions have been found to be consistently related to cognitive switching within a WCST paradigm (Monchi et al., 2001; Simard et al., 2011). Greater activity was displayed in cognitive-control-related frontoparietal regions that support higher-level executive processing. However, these networks may be differentially engaged during executive tasks (Braver et al., 2009). To improve cognitive performance, the brain activation pattern in older adults showed involvement of the frontopolar cortex and insula, possibly recruited to support processing task demands thought to be sub-served by primary neural resources.

Increased frontal activation during word-matching confirms the frontal cortex’s contribution to semantic retrieval, selection, and control demands. It remains to be determined whether semantic executive functions are primarily sustained by the frontal cortex or by other regions. Within cognitive control networks, frontoparietal regions may be differentially engaged depending on executive control demands. For example, maintaining information over a period of time and selecting responses have been associated with more frontal than parietal engagement (Braver et al., 2003). Furthermore, the parietal regions seem to represent a convergence zone for many executive domains (Duncan, 2006). Many neuroimaging studies have emphasized the parietal cortex’s contribution to a wide range of tasks, including working memory (Derrfuss et al., 2005; Riis et al., 2008) and interference resolution (Zysset et al., 2007; Campbell et al., 2012). Conceivably, involvement of parietal regions may mechanistically underlie task performance maintenance in healthy aging.

The most relevant neural pattern was observed for intergroup comparisons when maintain rule was subtracted from switch rule (**Figure 3A**). Indeed, older adults recruited the inferior parietal region (BA 39/40), while younger adults recruited the posterior prefrontal region (junction of BAs 6, 8, and 44). This age-related shift in activation from anterior (frontal) to posterior (parietal) region reflects different patterns of activation in the two age groups, indicating the use of different brain networks to mediate task performance. An integrative theory of aging proposed by Park and Reuter-Lorenz (2009) suggests that a dynamic-adaptive, neuro-functional reorganization takes place in which the older brain builds neural scaffolds to engage more functional resources and to compensate for the insufficiency of available basic-neural resources. Based on the results of this study, older adults tend to recruit parietal regions as an alternate strategy to ensure successful behavioral performance avoiding limited frontal resources. Furthermore in older adults, activity in the left inferior parietal region increased with a correct response and with a decreased response speed, whereas younger adults showed an inverse behavioral pattern (**Figure 3B**). This region-dependent pattern of activation and performance suggests that involvement of the inferior parietal region is necessary for older adults as another form of neuro-functional reorganization to maintain processing efficiency during a word-matching task.

In this context, activation in parietal regions can be considered as a more straightforward form of older-brain neural scaffold in response to neural insult and to age-related changes in frontal regions (Reuter-Lorenz and Cappell, 2008; Peelle et al., 2013). According to recent studies (Harding et al., 2015; Grady et al., 2016), the age-related changes in functional connectivity between frontal and parietal nodes are relevant in task performance, since greater neural recruitment in this cognitive control network co-occurs with greater improvements in executive processing. Those studies described patterns reflecting the neuro-functional adaptability of neural resources in healthy aging within the frontoparietal network, with a decrease in age-related parietal structural changes. This suggests that one of many mediating factors for neural resource reallocation is a cognitive control ability that enhances the development of dynamic neuro-functional reorganization trajectories that compensate for changes in the aging brain (Reuter-Lorenz and Cappell, 2008). These results provide some evidence for the HAROLD phenomenon in parietal regions (Cabeza, 2002; Grady et al., 2002; Angel et al., 2010). More interestingly, despite a decline in cognitive control processing in healthy aging, increasing reliance on parietal regions may be a means by which to functionally and cognitively cope with limited frontal resources. This possibility is supported by a recent observation that the aging brain is widely involved in executive control of resources during highly process-dependent tasks (Bouazzaoui et al., 2014). Nevertheless, the lack of correlation between behavioral performance and the right posterior prefrontal cortex implies that this region is less critical to successful executive processing in semantic tasks than is the inferior parietal region, which is associated with executive semantic processing (Noonan et al., 2013; Peelle et al., 2013).

In summary, executive functions have been found to support semantic performance, with increased reliance on neural substrates that sustain the demand for higher executive control; these substrates include the inferior parietal region, which is correlated with age-related performance maintenance in older age. However, it seems reasonable that changes in executive functional organization in healthy aging underlie very different executive abilities, and by extension, might predict distinct cognitive profiles preferentially sustained by distinct neural substrates as suggested by Hull et al. (2008). This may also explain why inter-individual variability in executive performance is more evident among older adults than younger ones (Logan et al., 2002; Braver and West, 2008). These possibilities are explored in the next section.

### Cognitive Control Profiles and Neuro-Functional Reorganization in Older Adults

A common finding on cognitive aging is that changes in cognitive function tend to be accompanied by changes in cognitive control processes in healthy elderly adults (Braver et al., 2001; Velanova et al., 2007; Mudar et al., 2015). Age-related executive function is supported by at least two executive components (updating and shifting), which make different contributions to task performance and probably depend on different neural patterns in healthy aging. The existence of different components within executive function (updating and shifting) indicates that executive functioning is organized in a non-unitary, multifaceted way in normal aging – and throughout the lifespan (Hull et al., 2008).

In accordance with our second prediction, we found two subgroups of older adults (updating-profile and shifting-profile) who showed different neuro-functional patterns depending on their executive abilities while performing equally well on the word-matching task (response times and correct responses). According to the flexible hub theory (Cole et al., 2013), a cognitive control network including the lateral prefrontal cortex, posterior parietal cortex and insula (which is known as the frontoparietal network) supports functional adaptation changes in healthy aging. Moreover, data supporting the theory indicates that age-related neuro-functional ability consists in flexibly coordinating cooperation between the cognitive control and language regions, to maintain task performance. Regarding the relevant functional regions and age-related executive changes, the findings reported herein support the hypothesis that older adults have developed a cognitive style that makes greater use of neural resources that mediate cognitive control of task performance.

Furthermore, several prior studies have highlighted activity changes in the frontoparietal network based on inter-individual differences in task performance. For example, Rypma et al. (2002) analyzed specific ROIs (dorsolateral, ventrolateral, posterior prefrontal cortex, and parietal cortex) and found that the frontoparietal network was associated with maintenance performance. In that study, older participants with slow response times showed bilateral activation in the dorsolateral prefrontal cortex, whereas older participants who responded faster showed widespread bilateral parietal cortex activation. Moreover, Gevins and Smith (2000) found differences in the involvement of frontal and parietal regions based on their participants’ cognitive ability. In their study, older adults with higher verbal ability showed greater activation in parietal regions, while those with lower verbal ability exhibited more activation in frontal regions.

These findings suggest that individual differences in performance may be due to distinct functional changes. In the same vein, Peelle et al. (2013) found differences in the involvement of frontal and parietal regions depending on older participants’ cognitive ability. They reported that older adults with higher cognitive function showed greater activation in parietal regions, while those with lower function exhibited more activation in frontal regions. These group differences suggest that older participants with more cognitive resources are able to adopt a strategy that involves larger, more distributed brain areas, while those with fewer resources use a strategy based on specific frontal regions.

Similarly, our results support this assumption since different patterns of neuro-functional reorganization in the frontoparietal network were found to relate to the elderly participant’s cognitive control profile. The updating-profile group improved their behavioral performance during maintain rule with more reliance on the dorsolateral prefrontal region (**Figure 5B**), which the shifting-profile group did not. Conversely, the inferior parietal region was more relevant for successful performance in the shifting-profile participants than for the updating-profile group’s performance during switch rule (**Figure 6B**). This result is of considerable importance, since it provides evidence for changes in executive process organization in healthy aging. As proposed by Noonan et al. (2013), the executive control over semantic processing underlies a flexible neural network including the bilateral posterior prefrontal cortex and the left inferior parietal cortex. Disruption of one region results in shift activation in other parts of the network. Hence, a healthy aging brain recruits more than one pathway to preserve cognitive performance.

## Conclusion

Age-differences in fronto-parietal region involvement during executive processing in a word-matching task are associated with behavioral performance maintenance by recruiting the inferior parietal region when executive frontal resources are in high demand. This greater reliance on the inferior parietal region by older adults could be interpreted as a neuro-functional change reflecting age-associated mechanistic differences in the executive control system, which are known to be dynamic in healthy aging. A distinction between frontal and parietal regions was also observed in older adults, which was related to cognitive control profile. This finding appears to be consistent with changes in executive process organization in healthy aging as proposed by Hull et al. (2008) and Adrover-Roig and Barceló (2010). Thus, diversity in executive control system serves to adapt older brain to different functional changes. This characterizes older people in terms of frontal and parietal network utilization for more proficient executive semantic processing, as assessed by a word-matching task. The relationships between changes in brain activity and in executive ability in healthy aging are relevant to future cognitive aging studies and more specifically to brain-injured individuals with semantic aphasia as well as executive dysfunction. We acknowledge that the relatively small number of elderly participants included in our second study represents a limitation; the study should be replicated with a larger sample. Even so, as discussed, our findings are in accordance with various previous studies.

## Author Contributions

Study conception and design: IM, MW, OM, J-SP, and YJ. Acquisition of Data: IM and YJ. Analysis and interpretation of data: IM, MA, BP, J-SP, and YJ. Drafting of manuscript: IM and YJ. Critical revision: IM, MA, BP, MW, JA, and YJ.

## Conflict of Interest Statement

The authors declare that the research was conducted in the absence of any commercial or financial relationships that could be construed as a potential conflict of interest.

## Footnotes

1 www.fmrib.ox.ac.uk/fsl
