# Supplementary material for: Age-Related Shift in Neuro-Activation during a Word-Matching Task
Source: Front Aging Neurosci. 2017 Aug 10;9:265. doi: 10.3389/fnagi.2017.00265 (PMC5554371; doi:10.3389/fnagi.2017.00265)
Supplement: Supplementary file 1 [file Table_1.docx]

Supplementary Material

**Age-Related Shift in Neuro-activation During a Word-Matching Task**

Ikram Methqal^1,2*^, Jean-Sebastien Provost^3^, Maximiliano A. Wilson^4^, Oury Monchi^5^, Mahnoush Amiri^1^, Basile Pinsard^2^, Jennyfer Ansado^6^, Yves Joanette^1,2^

^1^Laboratory of Communication and Aging, Institut Universitaire de Gériatrie de Montréal, Montreal, QC, Canada

^2^Faculty of Medicine, University of Montreal, QC, Canada

^3^Helen Wills Neuroscience Institute, University of California, Berkeley, Berkeley, CA, United States

^4^Centre de recherche CERVO - CIUSSS de la Capitale-Nationale et Département de réadaptation, Université Laval, Québec City, QC, Canada

^5^ Hotchkiss Brain Institute, University of Calgary, Calgary, AB, Canada

^6^ Department of Psychology, Université du Québec en Outaouais, Gatineau, QC, Canada.

***Correspondence:**Ikram Methqal
[ikrammethqal@gmail.com](mailto:ikrammethqal@gmail.com)

# Supplementary Tables

**Table S1| Maintain rule minus control matching.**

|  |  | **MNI peak (mm)** | | | | |  | | | | |
| --- | --- | --- | --- | --- | --- | --- | --- | --- | --- | --- | --- |
| **Cluster** | **Anatomical areas** | **x** | | **y** | **z** | **Z score** | **Voxels** | | | | |
|  | **Younger** |  | |  |  |  |  | | | | |
| 1 | Right dorsolateral prefrontal cortex (area 9/46) | 52 | | 31 | 27 | 4.75 | 11013 | | | | |
|  | Right insula (area 41) | 33 | | 26 | 1 | 4.38 |  | | | | |
| 2 | Left inferior parietal (area 39) | –23 | | –67 | 36 | 4.98 | 26572 | | | | |
|  | Right superior parietal cortex (area 7**)** | | 35 | –59 | 41 | 4.43 |  | | | | |
|  | Left superior parietal cortex (area 7) | | –21 | –65 | 47 | 4.25 |  | | | | |
| 3 | Left posterior prefrontal cortex (junction of 6, 8, and 44) | –46 | | 10 | 27 | 5.4 | 36766 | | | | |
|  | Left ventrolateral prefrontal cortex (area 44/45) | –42 | | 24 | 18 | 5.25 |  | | | | |
|  | Left lateral premotor cortex (area 6) | –37 | | 5 | 28 | 5.14 |  | | | | |
|  | Left insula (area 41) | –33 | | 22 | –2 | 4.42 |  | | | | |
|  | **Older** |  | |  |  |  |  | | | | |
| 1 | Left inferior temporal cortex (area 37, 20) | –51 | | –49 | –21 | 3.61 | | | 17812 | | |
|  | Left occipital cortex (area 18) | –27 | | –97 | –14 | 3.47 | | |  | | |
|  | Right cerebellum | 58 | | 20 | 21 | 3.32 | | |  | | |
| 2 | Right dorsolateral prefrontal cortex (area 9/46) | 52 | | 31 | 26 | 5.19 | | | 20501 | | |
|  | Right insula (area 41) | 31 | | 25 | 0 | 3.6 | | |  | | |
|  | Left insula (area 41) | –31 | | 21 | 0 | 3.57 | | |  | | |
|  | Right posterior prefrontal cortex (junction of 6, 8, and 44) | 52 | | 16 | 26 | 3.51 | | |  | | |
| 3 | Left dorsolateral prefrontal cortex (area 9/46) | –43 | | 15 | 25 | 5.57 | | | 40170 | | |
|  | Left posterior prefrontal cortex (junction of 6, 8, and 44) | –48 | | 17 | 31 | 5.03 | | |  | | |
|  | Left ventrolateral prefrontal cortex (area 44/45) | –41 | | 23 | 19 | 4.59 | | |  | | |
|  | Left lateral premotor cortex (area 6) | –40 | | 3 | 28 | 4.54 | | |  | | |
| 4 | Left inferior parietal cortex (area 40) | –30 | | –58 | 38 | 5.53 | | 40319 | | |  |
|  | Right superior parietal cortex (area 7) | 37 | | –64 | 48 | 5.05 | | | |  | |
|  | Left superior parietal cortex (area 7) | –31 | | –72 | 49 | 4.61 |  | | | | |
