# Supplementary material for: Age-Related Shift in Neuro-Activation during a Word-Matching Task
Source: Front Aging Neurosci. 2017 Aug 10;9:265. doi: 10.3389/fnagi.2017.00265 (PMC5554371; doi:10.3389/fnagi.2017.00265)
Supplement: Supplementary file 2 [file Table_2.docx]

Supplementary Material

**Age-Related Shift in Neuro-activation During a Word-Matching Task**

Ikram Methqal^1,2*^, Jean-Sebastien Provost^3^, Maximiliano A. Wilson^4^, Oury Monchi^5^, Mahnoush Amiri^1^, Basile Pinsard^2^, Jennyfer Ansado^6^, Yves Joanette^1,2^

^1^Laboratory of Communication and Aging, Institut Universitaire de Gériatrie de Montréal, Montreal, QC, Canada

^2^Faculty of Medicine, University of Montreal, QC, Canada

^3^Helen Wills Neuroscience Institute, University of California, Berkeley, Berkeley, CA, United States

^4^Centre de recherche CERVO - CIUSSS de la Capitale-Nationale et Département de réadaptation, Université Laval, Québec City, QC, Canada

^5^ Hotchkiss Brain Institute, University of Calgary, Calgary, AB, Canada

^6^ Department of Psychology, Université du Québec en Outaouais, Gatineau, QC, Canada.

***Correspondence:**Ikram Methqal
[ikrammethqal@gmail.com](mailto:ikrammethqal@gmail.com)

# Supplementary Tables

**Table S2| Switch rule minus control matching.**

|  |  | **MNI peak (mm)** | | | |  |
| --- | --- | --- | --- | --- | --- | --- |
| **Cluster** | **Anatomical areas** | **x** | **y** | **z** | **Z score** | **voxel** |
|  | **Younger** |  |  |  |  |  |
| 1 | Left occipital cortex (BA 18) | –34 | –90 | –15 | 5.59 | 13630 |
| 2 | Left superior parietal cortex (area 7) | –31 | –59 | 47 | 5.86 | 49418 |
|  | Left inferior parietal cortex (area 39) | –23 | –67 | 36 | 5.65 |  |
|  | Right superior parietal cortex (area 7) | 37 | –58 | 54 | 5.48 |  |
|  | Right inferior parietal cortex (area 39) | 33 | –60 | 40 | 5.18 |  |
| 3 | Left SMA (area 6) | –1 | 27 | 44 | 6.12 | 115940 |
|  | Left ventrolateral prefrontal cortex (area 44/45) | –42 | 23 | 20 | 6.06 |  |
|  | Right dorsolateral prefrontal cortex (area 9/46) | 45 | 29 | 24 | 5.8 |  |
|  | Right posterior prefrontal cortex (junction of 6, 8, and 44) | 39 | 18 | 49 | 5.76 |  |
|  | Anterior cingulate cortex (area 32) | 5 | 32 | 35 | 5.71 |  |
| 4 | Left caudate nucleus (head) | –5 | 16 | –7 | 3.78 | 12257 |
|  | Right caudate nucleus (head) | 6 | –16 | 6 | 3.80 |  |
|  | **Older** |  |  |  |  |  |
| 1 | Right dorsolateral prefrontal cortex (area 9/46) | 50 | 30 | 30 | 5.47 | 54820 |
|  | Right frontopolar (area 10) | 35 | 57 | 4 | 5.09 |  |
|  | Left frontopolar (area 10) | –49 | 37 | –2 | 4.67 |  |
|  | Right insula (area 41) | 37 | 22 | –5 | 3.7 |  |
| 2 | Left posterior prefrontal cortex (junction of 6, 8, and 44) | –42 | 15 | 26 | 6.63 | 81135 |
|  | Right superior parietal cortex (area 7) | 39 | –63 | 49 | 5.72 |  |
|  | Left lateral premotor cortex (area 6) | –40 | 3 | 28 | 5.56 |  |
|  | Left SMA (area 6) | –2 | 25 | 43 | 5.04 |  |
|  | Right lateral premotor cortex (area 6) | 28 | 5 | 50 | 5.04 |  |
|  | Left insula (area 41) | –34 | 20 | 3 | 3.71 |  |
| 3 | Left inferior parietal cortex (area 40) | –30 | –58 | 38 | 5.53 | 143491 |
|  | Right inferior parietal cortex (area 40) | 42 | –63 | 43 | 5.47 |  |
