# Supplementary material for: Age-Related Shift in Neuro-Activation during a Word-Matching Task
Source: Front Aging Neurosci. 2017 Aug 10;9:265. doi: 10.3389/fnagi.2017.00265 (PMC5554371; doi:10.3389/fnagi.2017.00265)
Supplement: Supplementary file 3 [file Table_3.docx]

Supplementary Material

**Age-Related Shift in Neuro-activation During a Word-Matching Task**

Ikram Methqal^1,2*^, Jean-Sebastien Provost^3^, Maximiliano A. Wilson^4^, Oury Monchi^5^, Mahnoush Amiri^1^, Basile Pinsard^2^, Jennyfer Ansado^6^, Yves Joanette^1,2^

^1^Laboratory of Communication and Aging, Institut Universitaire de Gériatrie de Montréal, Montreal, QC, Canada

^2^Faculty of Medicine, University of Montreal, QC, Canada

^3^Helen Wills Neuroscience Institute, University of California, Berkeley, Berkeley, CA, United States

^4^Centre de recherche CERVO - CIUSSS de la Capitale-Nationale et Département de réadaptation, Université Laval, Québec City, QC, Canada

^5^ Hotchkiss Brain Institute, University of Calgary, Calgary, AB, Canada

^6^ Department of Psychology, Université du Québec en Outaouais, Gatineau, QC, Canada.

***Correspondence:**Ikram Methqal
[ikrammethqal@gmail.com](mailto:ikrammethqal@gmail.com)

# Supplementary Tables

**Table S3| Switch rule minus maintain rule**

|  |  | **MNI peak (mm)** | | | |  |
| --- | --- | --- | --- | --- | --- | --- |
| **cluster** | **Anatomical areas** | **x** | **y** | **z** | **Z score** | **voxel** |
|  | **Younger** |  |  |  |  |  |
| 1 | Left frontopolar cortex (area 10) | –34 | 57 | –7 | 6.31 | 15779 |
| 2 | Left occipital cortex (area 18) | –34 | –92 | –14 | 6.13 | 63839 |
| 3 | Left superior parietal cortex (area 7) | –47 | –51 | 45 | 7.04 | 67001 |
|  | Right superior parietal cortex (area 7) | 44 | –50 | 47 | 7.01 |  |
|  | Right inferior parietal cortex (area 40) | 46 | –45 | 40 | 6.63 |  |
| 4 | Anterior cingulate cortex (area 32) | 8 | 30 | 37 | 6.9 | 142136 |
|  | Right posterior prefrontal cortex (junction of 6, 8, and 44) | 32 | 14 | 43 | 6.76 |  |
|  | Right dorsolateral prefrontal cortex (area 9) | 44 | 41 | 29 | 6.59 |  |
|  | **Older** |  |  |  |  |  |
| 1 | Left superior parietal cortex (area 7) | –17 | –77 | 56 | 5.64 | 156442 |
|  | Left occipital cortex (area 18) | –25 | –98 | –13 | 5.59 |  |
|  | Left inferior parietal cortex (area 40) | –45 | –49 | 33 | 5.47 |  |
|  | Right superior parietal cortex (area 7) | 2 | –74 | 53 | 5.3 |  |
| 2 | Left SMA (area 6) | –3 | 6 | 61 | 6.03 | 183731 |
|  | Left dorsolateral prefrontal cortex (area 9/46) | –41 | 21 | 29 | 5.84 |  |
|  | Left frontopolar cortex (area 10) | –44 | 49 | 7 | 5.83 |  |
|  |  |  |  |  |  |  |
