# Supplementary material for: Age-Related Shift in Neuro-Activation during a Word-Matching Task
Source: Front Aging Neurosci. 2017 Aug 10;9:265. doi: 10.3389/fnagi.2017.00265 (PMC5554371; doi:10.3389/fnagi.2017.00265)
Supplement: Supplementary file 4 [file Table_4.docx]

Supplementary Material

**Age-Related Shift in Neuro-activation During a Word-Matching Task**

Ikram Methqal^1,2*^, Jean-Sebastien Provost^3^, Maximiliano A. Wilson^4^, Oury Monchi^5^, Mahnoush Amiri^1^, Basile Pinsard^2^, Jennyfer Ansado^6^, Yves Joanette^1,2^

^1^Laboratory of Communication and Aging, Institut Universitaire de Gériatrie de Montréal, Montreal, QC, Canada

^2^Faculty of Medicine, University of Montreal, QC, Canada

^3^Helen Wills Neuroscience Institute, University of California, Berkeley, Berkeley, CA, United States

^4^Centre de recherche CERVO - CIUSSS de la Capitale-Nationale et Département de réadaptation, Université Laval, Québec City, QC, Canada

^5^ Hotchkiss Brain Institute, University of Calgary, Calgary, AB, Canada

^6^ Department of Psychology, Université du Québec en Outaouais, Gatineau, QC, Canada.

***Correspondence:**Ikram Methqal
[ikrammethqal@gmail.com](mailto:ikrammethqal@gmail.com)

# Supplementary Tables

**Table S4| Maintain rule minus control matching.**

|  |  | **MNI peak (mm)** | | | |  |
| --- | --- | --- | --- | --- | --- | --- |
| **Cluster** | **Anatomical areas** | **x** | **y** | **z** | **Z score** | **voxel** |
|  | **Updating-profile** |  |  |  |  |  |
| 1 | Left posterior prefrontal cortex (junction of 6, 8, and 44) | –44 | 14 | 24 | 4.14 | 21251 |
|  | Left ventrolateral prefrontal cortex (area 47/12) | –50 | 37 | –4 | 4.09 |  |
|  | Left dorsolateral prefrontal cortex (area 9/46) | –41 | 18 | 32 | 4.03 |  |
|  | Left lateral premotor cortex (area 6) | –31 | 11 | 60 | 3.95 |  |
| 2 | Left cerebellum | –10 | –89 | –30 | 4.63 | 26118 |
|  | Right cerebellum | 9 | –89 | –32 | 4.44 |  |
|  | **Shifting-profile** |  |  |  |  |  |
| 1 | Left inferior parietal cortex (area 40) | –29 | –63 | 39 | 4.64 | 22400 |
|  | Left superior parietal cortex (area 7) | –28 | –63 | 51 | 3.39 |  |
| 2 | Left inferior temporal cortex (area 20) | –36 | –31 | –24 | 3.40 | 23651 |
