# Supplementary material for: Age-Related Shift in Neuro-Activation during a Word-Matching Task
Source: Front Aging Neurosci. 2017 Aug 10;9:265. doi: 10.3389/fnagi.2017.00265 (PMC5554371; doi:10.3389/fnagi.2017.00265)
Supplement: Supplementary file 5 [file Table_5.docx]

Supplementary Material

**Age-Related Shift in Neuro-activation During a Word-Matching Task**

Ikram Methqal^1,2*^, Jean-Sebastien Provost^3^, Maximiliano A. Wilson^4^, Oury Monchi^5^, Mahnoush Amiri^1^, Basile Pinsard^2^, Jennyfer Ansado^6^, Yves Joanette^1,2^

^1^Laboratory of Communication and Aging, Institut Universitaire de Gériatrie de Montréal, Montreal, QC, Canada

^2^Faculty of Medicine, University of Montreal, QC, Canada

^3^Helen Wills Neuroscience Institute, University of California, Berkeley, Berkeley, CA, United States

^4^Centre de recherche CERVO - CIUSSS de la Capitale-Nationale et Département de réadaptation, Université Laval, Québec City, QC, Canada

^5^ Hotchkiss Brain Institute, University of Calgary, Calgary, AB, Canada

^6^ Department of Psychology, Université du Québec en Outaouais, Gatineau, QC, Canada.

***Correspondence:**Ikram Methqal
[ikrammethqal@gmail.com](mailto:ikrammethqal@gmail.com)

# Supplementary Tables

**Table S5| Switch rule minus control matching**

|  |  | **MNI peak (mm)** | | | |  |
| --- | --- | --- | --- | --- | --- | --- |
| **Cluster** | **Anatomical areas** | **x** | **y** | **z** | **Z score** | **voxel** |
|  | **Updating-profile** |  |  |  |  |  |
| 1 | Right lateral premotor cortex (area 6) | 40 | –3 | 60 | 3.71 | 39673 |
|  | Right dorsolateral prefrontal cortex (area 9/46) | 30 | 42 | 11 | 3.66 |  |
| 2 | Left posterior prefrontal cortex (junction of 6, 8, and 44) | –42 | 12 | 29 | 4.45 | 44919 |
| 3 | Left cerebellum | –10 | –89 | –30 | 4.63 | 128473 |
|  | Right cerebellum | 9 | –89 | –32 | 4.44 |  |
|  | Left superior parietal cortex (area 7) | –10 | –69 | 60 | 4.03 |  |
|  | Right occipital cortex (area 18) | 30 | –92 | 14 | 3.96 |  |
|  | Left occipital cortex (area 18) | –25 | –99 | –13 | 3.95 |  |
|  | **Shifting-profile** |  |  |  |  |  |
| 1 | Right dorsolateral prefrontal cortex (area 9/46) | 50 | 34 | 29 | 4.72 | 20635 |
|  | Right lateral premotor cortex (area 6) | 33 | 17 | 52 | 3.61 |  |
| 2 | Left ventrolateral prefrontal cortex (area 44/45) | –41 | 26 | 13 | 4.4 | 40156 |
|  | Left posterior prefrontal cortex (junction of 6, 8, and 44) | –46 | 17 | 24 | 4.38 |  |
|  | Right posterior prefrontal cortex (junction of 6, 8, and 44) | 37 | 12 | 42 | 4.24 |  |
|  | Left frontopolar cortex (area 10) | -38 | 51 | 12 | 4.24 |  |
| 3 | Right superior parietal cortex (area 7) | 25 | –73 | 53 | 5.55 | 136037 |
|  | Left inferior parietal cortex (area 39/40) | –33 | –61 | 39 | 5.24 |  |
|  | Right cerebellum | 35 | -66 | -33 | 4.88 |  |
|  |  | | | | |  |
