# Supplementary material for: Age-Related Shift in Neuro-Activation during a Word-Matching Task
Source: Front Aging Neurosci. 2017 Aug 10;9:265. doi: 10.3389/fnagi.2017.00265 (PMC5554371; doi:10.3389/fnagi.2017.00265)
Supplement: Supplementary file 6 [file Data_Sheet_1.docx]

Supplementary Material

**Age-Related Shift in Neuro-activation During a Word-Matching Task**

Ikram Methqal^1,2*^, Jean-Sebastien Provost^3^, Maximiliano A. Wilson^4^, Oury Monchi^5^, Mahnoush Amiri^1^, Basile Pinsard^2^, Jennyfer Ansado^6^, Yves Joanette^1,2^

^1^Laboratory of Communication and Aging, Institut Universitaire de Gériatrie de Montréal, Montreal, QC, Canada

^2^Faculty of Medicine, University of Montreal, QC, Canada

^3^Helen Wills Neuroscience Institute, University of California, Berkeley, Berkeley, CA, United States

^4^Centre de recherche CERVO - CIUSSS de la Capitale-Nationale et Département de réadaptation, Université Laval, Québec City, QC, Canada

^5^ Hotchkiss Brain Institute, University of Calgary, Calgary, AB, Canada

^6^ Department of Psychology, Université du Québec en Outaouais, Gatineau, QC, Canada.

***Correspondence:**Ikram Methqal
[ikrammethqal@gmail.com](mailto:ikrammethqal@gmail.com)

# Supplementary Data

***Word selection procedure***

The experimental task required participants to sort words according to different levels of semantic relatedness. In order to construct the task, 169 stimulus words were chosen from the databases created by Dubois and Reshe-Rigon (1995*)* and Léger et al., (2008). The category ‘animals’ was selected (subcategories: birds, insects, quadrupeds, and fish). Before the final stimuli were presented to participants, a pilot study was carried out in which 40 volunteers (20 younger, M = 26.92 years; SD = 6.02 and 20 older, M = 60 years, SD = 5.75) were asked to identify the items they had never heard before in a list of words. Based on this assessment, items that were identified as unknown by three or more participants were excluded.

Afterwards, two types of semantic relatedness were also measured: typicality relatedness and functional relatedness. Two groups of 20 participants each (20 younger and 20 older) were asked to estimate the extent to which these words represented a category on a 7-point scale (1 = least typical; 7 = most typical). First, the typical and atypical words were selected following the consensus obtained by the two groups (Typical: younger: mean = 5.67, SD = 0.09; older: mean = 5.58, SD = 0.10; Atypical: younger: mean = 3.77, SD = 0.1; older: mean = 3.75, SD = 0.99). Then, from this selection, the moderately and highly typical words were selected and divided into two lists. The highly typical words were chosen as target words and presented to the same sample of participants in order to establish a list of functionally related words. For the last step in stimulus selection, a similar pilot study assessed the extent to which five words (databases of Nelson et al., 2004 and De Deyne and Storms, 2008) were strongly associated with each highly typical target word. The final list contained 40 highly typical words, 40 moderately typical words, 40 atypical words and 40 functionally related words. None of the pilot participants were invited to participate in the experimental word-matching task. All stimuli were controlled for frequency, word length and imageability with the French lexical database Lexique 3, from Paris Descartes University, France (New et al., 2005; [www.lexique.org](http://www.lexique.org/)). No significant difference was found between the three reference words (moderately typical; atypical, and functional) in terms of lexical frequency [moderately typical (M = 4.98, SD = 3.73), atypical (M = 4.07, SD = 4.90), and functional (M= 5.95, SD = 7.36); F(2,117) = 1.15; p = 0.32]; word length [moderately typical (M = 6.60, SD = 1.49), atypical (M = 6.60, SD= 1.53), and functional (M = 6.80, SD = 1.34); F(2,117) = 0.25; p = 0.78] and imageability [moderately typical (M = 5.19, SD = 1.01), atypical (M = 4.86, SD = 1.04), and functional (M = 5.23, SD = 1.33); F(2,117) = 1.24; p = 0.29].
